# Healthcare Costs Associated with an Adequate Intake of Sugars, Salt and Saturated Fat in Germany: A Health Econometrical Analysis

**DOI:** 10.1371/journal.pone.0135990

**Published:** 2015-09-09

**Authors:** Toni Meier, Karolin Senftleben, Peter Deumelandt, Olaf Christen, Katja Riedel, Martin Langer

**Affiliations:** 1 Institute of Agricultural and Nutritional Sciences, Martin Luther University Halle-Wittenberg, Halle (Saale), Germany; 2 BRAIN Biotechnology Research And Information Network AG, Zwingenberg, Germany; University Hospital Oldenburg, GERMANY

## Abstract

Non-communicable diseases (NCDs) represent not only the major driver for quality-restricted and lost life years; NCDs and their related medical treatment costs also pose a substantial economic burden on healthcare and intra-generational tax distribution systems. The main objective of this study was therefore to quantify the economic burden of unbalanced nutrition in Germany—in particular the effects of an excessive consumption of fat, salt and sugar—and to examine different reduction scenarios on this basis. In this study, the avoidable direct cost savings in the German healthcare system attributable to an adequate intake of saturated fatty acids (SFA), salt and sugar (mono- & disaccharides, MDS) were calculated. To this end, disease-specific healthcare cost data from the official Federal Health Monitoring for the years 2002–2008 and disease-related risk factors, obtained by thoroughly searching the literature, were used. A total of 22 clinical endpoints with 48 risk-outcome pairs were considered. Direct healthcare costs attributable to an unbalanced intake of fat, salt and sugar are calculated to be 16.8 billion EUR (CI95%: 6.3–24.1 billion EUR) in the year 2008, which represents 7% (CI95% 2%-10%) of the total treatment costs in Germany (254 billion EUR). This is equal to 205 EUR per person annually. The excessive consumption of sugar poses the highest burden, at 8.6 billion EUR (CI95%: 3.0–12.1); salt ranks 2^nd^ at 5.3 billion EUR (CI95%: 3.2–7.3) and saturated fat ranks 3^rd^ at 2.9 billion EUR (CI95%: 32 million—4.7 billion). Predicted direct healthcare cost savings by means of a balanced intake of sugars, salt and saturated fat are substantial. However, as this study solely considered direct medical treatment costs regarding an adequate consumption of fat, salt and sugars, the actual societal and economic gains, resulting both from direct and indirect cost savings, may easily exceed 16.8 billion EUR.

## Introduction and Objective of the Study

Diet-related non-communicable diseases represent in Germany and in other industrialized countries an important cost factor in healthcare systems. Besides this, western dietary patterns involving an overconsumption of fatty, sugary and salty foods proliferate in many emerging and developing economies, which results in an increased prevalence of degenerative diseases [1, 2]. While until the middle of the 20^th^ century one of the core tasks of food and health policies was to ensure a quantitatively sufficient food supply, the main task today is to align unbalanced food and nutrient supplies in accordance with official dietary recommendations. Moreover, western dietary patterns are criticised for being a major driver of global environmental change, linked to an unsustainable use of limited resources, the depletion of ecosystem services and a critical transgression of planetary boundaries [3–6].

Bloom et al. (2011) stated that the global costs of non-communicable diseases (e.g. obesity, diabetes, cancer, cardiovascular diseases) will rise from 6.2 trillion US$ in the year 2010 to 17 trillion US$ in the year 2030 [7]. This forecast is substantiated by the results of the Global Burden of Disease Study 2010, which confirmed a global increase of non-communicable diseases in comparison to infectious diseases (from 1990 to 2010) [8]. Nevertheless, on a global level there are not currently available any reliable data on the extent to which healthcare expenditures on non-communicable diseases are related to dietary factors.

The direct medical costs of obesity worldwide were estimated in a review by Withrow and Alter (2010) to account for between 0.7% and 2.8% of a country’s total healthcare expenditure—depending on the corresponding obesity burden and the modelling approach applied in the underlying studies [9]. The American Diabetes Association calculated the total direct and indirect diabetes-related costs in the United States to be 174 billion US$, including 116 billion US$ in excess medical expenditure and 58 billion in reduced national productivity [10]. Covering a broad set of intervention measures (lifestyle, medication, etc.), the UnitedHealth Group estimated that, from 2011 to 2020, 25 billion US$ are saveable in the United States per year in terms of prediabetes and diabetes-related costs—with a federal share of 58% [11]. The direct medical costs of cardiometabolic risk factors were estimated by Sullivan et al. (2007) to be 80 billion US$ in the United States in 2000/2002—differentiating between several insurance payers [12]. Barnard et al. (1995) estimated the direct medical treatment costs attributable to meat consumption in the United States to range between 28.6 and 61.4 billion US$ [13]. For Canada, Joffres et al. (2007) [14] calculated direct medical cost savings of approximately $430 million per year associated with a balanced intake of sodium, which represents 18% of all hypertension-related costs.

For Switzerland, the economic burden of overweight/obesity was examined by Schmid et al. (2005), accounting for 2.3%-3.5% of total healthcare expenditure (2.1–3.2 billion CHF) [15]. For cigarette smoking in Germany, Welte et al. (2000) calculated for the year 1993 direct medical costs of 9.3 billion German marks (equivalent of 4.8 billion EUR using the official exchange rate of the year 1999) and indirect costs of 24.6 billion German marks (equivalent of 12.6 billion EUR)—totalling 33.8 billion German marks (equivalent of 17.3 billion EUR) [16]. Taking into account the total direct treatment costs of all diseases covered by the official Federal Health Monitoring in Germany, these rose nominally from the year 1992 to 2012 from 158 to 300 billion EUR, with an increasing share of GDP from 9.4% to 10.9% [17]. For Western Germany, Arab-Kohlmeier et al. (1993) had shown that diet-related risk factors contributed to 30% of all direct and indirect healthcare expenditures. Based on the reference year 1990, these amounted to a total of 83.5 billion German marks, which is the equivalent of 42.7 billion EUR (using the official exchange rate of the year 1999). While the direct costs were estimated to be 47.3 billion German marks (equivalent of 24.2 billion EUR), the indirect costs accounted for 36.2 billion German marks (equivalent of 18.5 billion EUR) [18]. As more recent data concerning the nutrition-related disease burden and corresponding costs in the German healthcare system have not been published, this study is intended to answer the following questions:

1. To what extent does the current intake of mono- & disaccharides (MDS), salt and saturated fatty acids (SFA) contribute to the direct treatment costs of related non-communicable diseases in Germany?
2. Which direct cost savings can be expected on the national level if the current dietary intake of the considered risk factors were closer to or in line with official dietary recommendations?
3. How reliable are the generated results in the face of uncertainty and sensitivity checks?

The material and methods section describes the separate methodological steps used to answer these questions. The basis for the health economic analysis were, on the one hand, the disease-specific healthcare cost data from the official Federal Health Monitoring [19] and disease-related risk factors, obtained by thoroughly searching the literature. On the other hand, the National Nutrition Survey II from the year 2006 was used—the most up-to-date and representative survey of food and nutrient intake in Germany [20, 21]. Table A in S1 File (supporting information) gives an overview of the intake levels and corresponding recommendations according to gender and age group. Particularly in the case of MDS and SFA, an excessive consumption is obvious. Depending on the survey sample of the National Nutrition Survey II ([20] n = 15,371, [21] n = 13,753), an overconsumption in terms of salt was observed for both men and women using data from [20], but only for men using data from [21].

### Sodium / salt as a risk factor

An excessive intake of salt is associated with a broad range of non-communicable diseases, such as cardiovascular diseases [22, 23], cancer [24, 25] and osteoporosis [26, 27]. According to a comparative risk analysis, Mozaffarian et al. (2014) calculated that, on a global level, 1.65 million deaths per year are attributable to an excessive intake of salt [23]. From 2003 to 2011, He et al. (2013) observed a declined systolic and diastolic blood pressure in the UK, of-4.18 mm Hg (95%CI −5.18 to −3.18) and −2.06 mm Hg (−2.67 to −1.45) respectively, as a result of diminished salt intake [22]. Salt sensitivity is a measure of how blood pressure responds to salt intake. According to the Federal Institute of Risk Assessment, 20–30% of the German population and 50% of persons with high blood pressure are salt-sensitive [28]. Moreover, overweight/obesity and the metabolic syndrome are associated with an increased salt sensitivity [29–31]. Within the Global Burden of Disease Study 2010, it was calculated that, in 2010, a total of 11.6 million years lived with disability (YLD) were attributable to an excessive intake of salt in Germany (equivalent of 0.8% of all YLD) [32].

### Saturated fat as a risk factor

According to the evidence-based guidelines for fat of the German Nutrition Society, there is convincing evidence that saturated fatty acids (SFA) elevate the risk of dyslipoproteinemia, with an increase of LDL cholesterol, and also possible evidence of a risk of cardiovascular diseases [33, 34]. With possible evidence a substitution of SFAs by PUFAs leads to a decreased risk of ischemic heart diseases [34]. No effect regarding an incline or decline is detectable with convincing evidence for hypertension as well as no effect with possible evidence for type II diabetes and cancer (with the exception of breast cancer). The risk of breast cancer is increased as a result of an elevated intake of SFA [33,35]. Further, it should be kept in mind that SFAs represent almost 16% of the total fat consumed in Germany (recommendation: 10%) and that total fat consumption is above the recommendation (see Table A in S1 File). For this reason, an indirect causal relationship between the excessive intake of SFAs and the prevalence of obesity must be assumed [36, 37]. A body mass index in between 25 and 29.9 (overweight) and of 30 and higher (obesity), respectively, correlates with the development of different forms of cancer [38], chronic obstructive pulmonary disease (COPD) [39] and Alzheimer disease [40].

### Sugar (mono- & disaccharides) as a risk factor

According to the evidence-based guidelines for carbohydrates of the German Nutrition Society, no direct correlation exists between the intake of mono- and disaccharides (MDS) and the prevalence of clinical endpoints. However, there is convincing evidence that the intake of sugar-sweetened beverages correlates with developing overweight/obesity, diabetes type II and the metabolic syndrome [41]. Further, there is some indication (possible evidence) of a relationship between the excessive intake of MDS and pancreatic cancer [42,43], colon cancer [44] as well as chronic kidney disease as a comorbidity of diabetes, hypertension and kidney stones [45]. In a cross-sectional study, Basu et al. (2013) detected a 1.1% increase (CI95%: 0.48%-1.7%) in the prevalence of diabetes as a result of the extra uptake of 150 kcal sugar per person per day, which is the equivalent of 35g of sugar [46]. The substantial impact of sugar consumption on dental caries and other diseases of the hard tissues of teeth is described in [47–49].

## Materials and Methods

### Study selection

A literature search in several databases (PUBMED, WEB OF SCIENCE, Cochrane Database of Systematic Reviews) was conducted for the period from 1950 until 2014 with regard to the risk factors considered (mono- & disaccharides, salt/sodium, saturated fatty acids) and associated diseases. The specific search terms used in the search queries and related hits can be found in the S1 File (Table B). The archives of the WCRF, the WHO and the German Nutrition Society (DGE) were also searched and experts contacted for further relevant papers. Abstracts and unpublished studies were not included. The procedure of the literature search and the study selection is summarized in Table 1.

**Table 1:** Results of the literature search and the study selection.

Database search results	Hits
**WEB OF SCIENCE**	**1765**
… sugar (mono- & dicarbohydrates)	146
… fat (saturated fatty acids)	190
… sodium / salt	1429
**PubMed**	**1023**
… sugar (mono- & dicarbohydrates)	87
… fat (saturated fatty acids)	206
… sodium / salt	730
**Cochrane reviews**	**753**
… sugar (mono- & dicarbohydrates)	135
… fat (saturated fatty acids)	512
… sodium / salt	106
**SUM (including double counts)**	**3541**
… studies excluded on basis of title and/or abstract (clearly did not meet inclusion criteria)	3468
… studies included from archives and institutional websites (WCRF, WHO, DGE)	4
… potentially relevant studies	77
… studies excluded because, after in-depth examination, they did not meet the inclusion criteria	63
… **studies included in the econometrical analysis**	**14**

Finally, of the 77 studies identified as relevant 63 were excluded, because after in-depth examination they did not meet the inclusion criteria. A full list of these studies and the reasons for their exclusion can be found in the S1 File (Table C). Whenever possible, econometrical data concerning current intake and prevalence levels in Germany based on randomized and controlled meta-studies were used. Within the meta-studies, intervention studies were favoured over non-controlled intervention studies, cohort studies and case control studies (in this descending order)—following the level of evidence classification of the Scottish Intercollegiate Guidelines Network [50–52]. The following levels of evidence are distinguished here (in descending order):

- Ia meta-analysis of randomized, controlled intervention studies
- Ib randomized, controlled intervention studies
- Ic non-randomized / non-controlled intervention studies
- IIa meta-analysis of cohort studies
- IIb cohort studies
- IIIa meta-analysis of case-control studies
- IIIb case-control studies
- IV non-analytical studies, expert opinions, consensus papers

Studies with an evidence level of IV were excluded from the econometrical analysis.

### Scope

As regards the risk factors considered, the direct medical treatment costs of associated diseases and comorbidities were calculated. Data from the official Federal Health Monitoring were used as the basis for the direct healthcare costs [19]. Here the direct treatment costs for 137 disease groups are covered for the years 2002, 2004, 2006 and 2008. Direct costs comprise all the costs that arise directly as a result of treatment, prevention, medication, physician visits and hospital stays. Indirect costs were omitted from this study; these are productivity losses (lost wages) caused by the respective disease through time off work, early retirement and premature death. Using the population attributable risk (PAR), the direct medical treatment costs were then attributed to corresponding risk factors, resulting from an overconsumption of MDS, sodium/salt and SFA. If the underlying studies provided only the relative risk and/or odds ratios, these were converted to the PAR using the following formula (according to [53]):

$$PAR=1-\frac{1}{p(RR-1)+1}$$

PAR … population attributable risk

p … prevalence

RR … relative risk

The following formula was used to convert odds ratios to relative risks (according to [54]):

$$RR=\frac{OR}{1-p_{o}+(p_{o}*OR)}$$

OR … odds ratio

p_0_ … base risk

RR … relative risk

Due to the goal of the study, no calculation was performed of risk factor- and disease-specific *years of life lost due to premature death* (YLL), *years lived with disabilit*y (YLD) or *disability-adjusted life years* (DALYs). If the data used from the official Federal Health Monitoring [19] did not provide sufficiently detailed information on the disease level (but only on the disease group level), the related treatment costs were allocated using data from the more explicit dataset concerning treated and released hospital patients [55]. With 1730 distinct diseases, this dataset is more precise than the former one, which solely covers 137 diseases/disease groups. The following disease costs were approximated by using this allocative approach (Table 2):

**Table 2:** Diseases with treatment cost allocation according to treated and released hospital patients (based on [55]).

Disease	ICD10 code
Peripheral vascular disease	I70,I73
Malignant neoplasm of oesophagus	C15
Malignant neoplasm of gallbladder	C23
Malignant neoplasm of corpus uteri	C54
Malignant neoplasm of ovary	C56
Malignant neoplasm of kidney, renal pelvis, ureter	C64-C66
Chronic obstructive pulmonary disease	J40-J44, J47
Chronic kidney disease	N02–05, N15, N20-N23
Coxarthrosis [arthrosis of hip]	M16
Gonarthrosis [arthrosis of knee]	M17

### Scenario analysis

The scenario analysis looked into a stepwise reduction of the excessive consumption of the risk factors considered. While in the standard scenario a 100% reduction was assumed, in the scenario analysis the economic effect of a 10%, 30%, 50% and 70% reduction was examined. Here, a linear dependence (a linear dose-response-function) between risk factor and corresponding treatment cost was assumed, meaning that e.g. a 10% reduction of the excessive sugar consumption would lead to a 10% decline of related disease burden and healthcare costs. Other dose-response-functions were not available.

### Sensitivity, uncertainty and completeness analysis

As the results of this study rely on distinct data sources with different evidence levels and different study designs sensitivity and uncertainty checks were conducted to prove the validity of the results. Firstly, the validity was gauged by analysing the 95% confidence interval for all statistical effect sizes considered (RR, OR, PAR). In the case of the data extracted from IHME (2014), the 95% probability interval was used, as the results of IHME (2014) are not based on a frequentist, but on a Bayesian statistical data analysis [32]. Secondly, in order to reflect different levels of salt sensitivities [56], which result in varying effects of salt-related diseases, a salt sensitivity of 50% in case of hypertensive-associated diseases according to BfR (2008) was examined separately [28]. Thirdly, the completeness of the study in terms of the articles and dose-response relationships included was ensured by systematically searching the literature at the beginning of the analysis (see Table 1).

## Results

**Fig 1:**
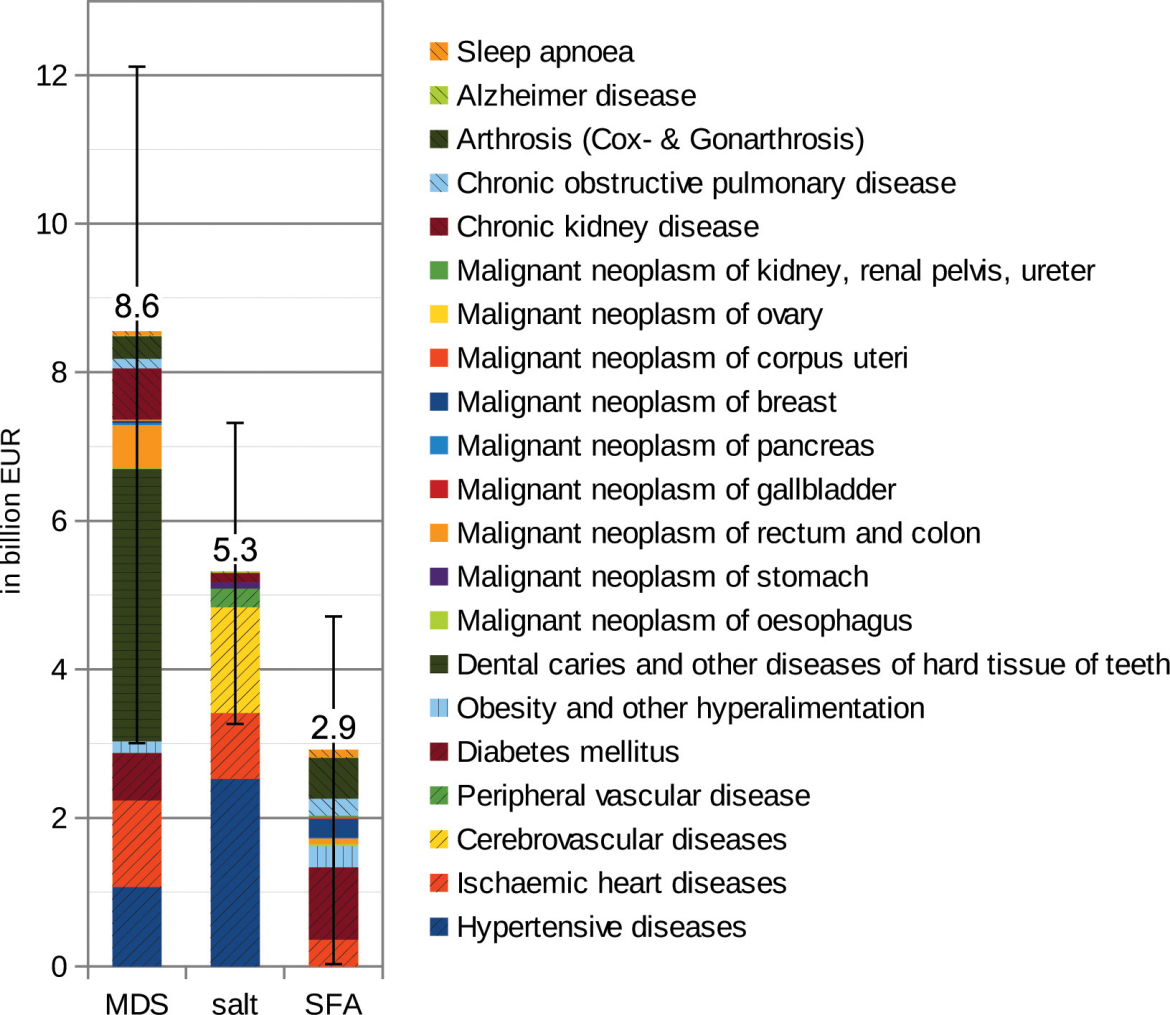
Healthcare costs associated with an overconsumption of MDS, salt and SFA (incl. 95% confidence interval).

With an adequate intake of mono- & disaccharides (MDS), salt and saturated fatty acids (SFA), annual healthcare cost savings are calculated to be 16.8 billion EUR (CI95%: 6.3–24.1 billion EUR), which represents 7% (CI95% 2%-10%) of the total medical treatment costs in the year 2008 (254 billion EUR) in Germany. This is equal to 205 EUR per person annually (CI95%: 77–294). The overconsumption of sugar imposes the highest burden, at 8.6 billion EUR (CI95%: 3.0–12.1)—mainly due to the costs of treating caries and other diseases of the hard tissues of teeth, hypertensive and cardiovascular diseases, diabetes mellitus, rectum and colon cancer as well as chronic kidney disease. The strongest impact on hypertensive, cardio- and cerebrovascular diseases came from an oversupply of sodium/salt, which alone resulted in 4.8 billion EUR of treatment costs. All in all, the overconsumption of salt leads to healthcare costs of 5.3 billion EUR (CI95%: 3.2–7.3). The unbalanced intake of saturated fatty acids was associated with 2.9 billion EUR (CI95%: 32 million—4.7 billion)—mainly due to the costs of treating diabetes mellitus, obesity, ischemic heart disease, chronic obstructive pulmonary disease and arthrosis (mainly mediated by overweight/obesity) (Fig 1). Obesity and other forms of hyperalimentation alone, triggered by an excessive consumption of MDS and SFA, accounted for direct medical treatment costs of 0.44 billion EUR (CI95%: minus 39 million-0.75 billion). Taking overweight/obesity-mediated comorbidities additionally into account, 3.6 billion EUR (CI95%: minus 0.24–4.5 billion) are attributable to an overconsumption of MDS and SFA, which represents 1.4% (CI95%: -0.1%-1.8%) of total healthcare expenditure. The highest uncertainty ratio—and, therefore, the lowest validity of the risk factors considered in terms of their impacts—was observed in the case of SFA (1.60), followed by MDS (1.06) and salt (0.76). The uncertainty ratio is defined as the CI95% range (CI95% max minus CI95% min) divided by the average mean.

Table 3 presents the total treatment and avoidable healthcare costs according to the risk factors and diseases analysed as well as related data sources and evidence levels. Table 4 gives an overview of corresponding population attributable and relative risks—including the 95% confidence intervals.

**Table 3:** Total and avoidable healthcare costs for the diseases considered resulting from a balanced intake of MDS, salt, SFA.

	ICD10 code	Total treatment costs 2008	Avoidable treatment costs resulting from a balanced intake of			Sources	Evidence level (EL)
			mono- & disaccharides	sodium / salt	saturated fatty acids		
		in million EUR					
Hypertensive diseases	I10–15	9,059	1,070			Dhingra et al. 2007 [41]	EL II b
				2,525		IHME 2014 [32]	Econometric model based on EL I, II, III
Ischemic heart diseases	I20–25	6,202	1,164			Dhingra et al. 2007 [41]	EL II b
				887		IHME 2014 [32]	Econometric model based on EL I, II, III
					362	Mensink et al. 2003 [37]	EL II a, III a
Cerebrovascular diseases	I60-I63, I65-I67, I69	7,788		1,425		IHME 2014 [32]	Econometric model based on EL I, II, III
Peripheral vascular disease	I70,I73	2,349		247		IHME 2014 [32]	Econometric model based on EL I, II, III
Diabetes mellitus	E10-E14	6,342	103			Basu et al. 2013 [46]	Econometric model based on EL II a
… overweight/obesity mediated			537		972	Schmid et al. 2004 [15]	Econometric model based on EC I, II, III
Obesity and other hyperalimentation	E65-E68	863	158			Dhingra et al. 2007 [41]	EL II b
					286	Schulz et al. 2002 [36]	EL II b
Dental caries and other diseases of the hard tissues of teeth	K02, K03	8,525	3,666			Moynihan, Kelly 2013 [47]	EL II a, EL IIIa
Malignant neoplasm of oesophagus	C15	281					
… overweight/obesity mediated			15		27	Arnold et al. 2014 [38]	EL Ia, IIa, IIIa
Malignant neoplasm of stomach	C16	513		91		IHME 2014 [32]	Econometric model based on EL I, II, III
Malignant neoplasm of rectum and colon	C18, C20	1,730	537			Bostick et al. 1994 [44]	EL IIb
… overweight/obesity mediated			40		73	Arnold et al. 2014 [38]	EL Ia, IIa, IIIa
Malignant neoplasm of gallbladder	C23	45					
… overweight/obesity mediated			1.3		2.4	Arnold et al. 2014 [38]	EL Ia, IIa, IIIa
Malignant neoplasm of pancreas	C25	462	22			Gallus et al. 2011 [43]	EL II a, III a
… overweight/obesity mediated			6.9		12	Arnold et al. 2014 [38]	EL Ia, IIa, IIIa
Malignant neoplasm of breast	C50	1,970			211	Sieri et al. 2007 [35]	EL II a
… overweight/obesity mediated			25		44	Arnold et al. 2014 [38]	EL Ia, IIa, IIIa
Malignant neoplasm of corpus uteri	C54	194					
… overweight/obesity mediated			11		21	Arnold et al. 2014 [38]	EL Ia, IIa, IIIa
Malignant neoplasm of ovary	C56	325					
… overweight/obesity mediated			1.4		2.6	Arnold et al. 2014 [38]	EL Ia, IIa, IIIa
Malignant neoplasm of kidney, renal pelvis, ureter	C64-C66	254					
… overweight/obesity mediated			8.8		16	Arnold et al. 2014 [38]	EL Ia, IIa, IIIa
Chronic kidney disease	N02–05, N15, N20-N23	1,232	689			Saldana et al. 2007 [45]	EL III b
				124		IHME 2014 [32]	Econometric model based on EL I, II, III
Chronic obstructive pulmonary disease	J40-J44, J47	4,646					
… overweight/obesity mediated			127		230	Behrens et al. 2014 [39]	EL II b
Coxarthrosis [arthrosis of hip]	M16	2,969					
… overweight/obesity mediated			67		121	Schmid et al. 2004 [15]	Econometric model based on EL I, II, III
Gonarthrosis [arthrosis of knee]	M17	3,762					
… overweight/obesity mediated			236		427	Schmid et al. 2004 [15]	Econometric model based on EL I, II, III
Alzheimer’s disease	G30	993					
… overweight/obesity mediated			7.5		14	Norton et al. 2014 [40]	Econometric model based on EL I, II, III
… hypertension mediated			8.0	19			
… diabetes mediated			0.5				
Sleep apnoea	G47.3	694					
… overweight/obesity mediated			53		96	Schmid et al. 2004 [15]	Econometric model based on EL I, II, III
Sum		**61,200**	**8,553**	**5,318**	**2,918**		

**Table 4:** Population attributable risks and relative risks according to risk factors and related diseases (incl. CI95%).

		Total treatment costs 2008	Population attributable risk resulting from an unbalanced intake of						Relative risk resulting from an unbalanced intake of					
	ICD10 code	in million EUR	mono- & disaccharides		sodium / salt		saturated fatty acids		mono- & disaccharides		sodium / salt		saturated fatty acids	
Hypertensive diseases	I10−15	9,059	11.81		27.87				1.13		1.39			
			-3.23-23.65		17.79-37.52				0.97-1.31		1.22-1.60			
Ischemic heart diseases	I20−25	6,202	18.76		14.31		5.84		1.23		1.17		1.06	
			4.38-30.20		8.82-19.79		4.41-7.27		1.05-1.43		1.10-1.25		1.05-1.08	
Cerebrovascular diseases	I60-I63, I65-I67, I69	7,788			18.30						1.22			
					11.38-24,84						1.13-1.33			
Peripheral vascular disease	I70,I73	2,349			10.52						1.12			
					6.32-14.68						1.07-1.17			
Diabetes mellitus	E10-E14	6,342	1.63						1.02					
			0.70-2.50						1.01-1.03					
… overweight/obesity-related			8.46				15.33		1.09				1.18	
			0.70-14.47				-2.78-25.88		1.01-1.17				0.97-1.35	
Obesity and other hyperalimentation	E65-E68	863	18.32				33.18		1.22				1.50	
			1.52-31.33				-6.03-56.01		1.02-1.46				0.94-2.27	
Dental caries and other diseases of the hard tissues of teeth	K02, K03	8,525	43.01						1.75					
			32.14-44.05						1.47-1.79					
Malignant neoplasm of oesophagus	C15	281												
… overweight/obesity mediated			5.39				9.77		1.06				1.11	
			3.77-6.87				6.83-12.44		1.04-1.07				1.07-1.14	
Malignant neoplasm of stomach	C16	513			17.65						1.21			
					-1.21-34.04						0.99-1.52			
Malignant neoplasm of rectum and colon	C18, C20	1,730	31.03						1.45					
			-13.64-58.16						0.88-2.39					
… overweight/obesity mediated			2.32				4.20		1.02				1.04	
			1.35-2.98				2.44-5.40		1.01-1.03				1.03-1.06	
Malignant neoplasm of gallbladder	C23	45												
… overweight/obesity mediated			2.96				5.37		1.03				1.06	
			0.15-4.98				0.26-9.02		1.00-1.05				1.00-1.10	
Malignant neoplasm of pancreas	C25	462	4.76						1.05					
			-6.38-14.53						0.94-1.17					
… overweight/obesity mediated			1.49				2.70		1.02				1.03	
			-0.19-2.93				-0.35-5.31		1.00-1.03				1.00-1.06	
Malignant neoplasm of breast	C50	1,970					10.71						1.12	
							1.96-18.03						1.02-1.22	
… overweight/obesity mediated			1.25				2.26		1.01				1.02	
			0.58-1.87				1.04-3.38		1.01-1.02				1.01-1.04	
Malignant neoplasm of corpus uteri	C54	194												
… overweight/obesity mediated			5.86				10.61		1.06				1.12	
			5.21-6.45				9.44-11.68		1.05-1.07				1.10-1.13	
Malignant neoplasm of ovary	C56	325												
… overweight/obesity mediated			0.44				0.79		1.00				1.01	
			-0.30-0.98				-0.55-1.78		1.00-1.01				0.99-1.02	
Malignant neoplasm of kidney, renal pelvis, ureter	C64-C66	254												
… overweight/obesity mediated			3.47				6.29		1.04				1.07	
			2.54-4.34				4.59-7.87		1.03-1.05				1.05-1.09	
Chronic kidney disease	N02−05, N15, N20-N23	1,232	55.89		10.04				2.27		1.11			
			28.54-72.75		5.78-15.48				1.40-3.67		1.06-1.18			
Chronic obstructive pulmonary disease	J40-J44, J47	4,646												
… overweight/obesity mediated			2.73				4.95		1.03				1.05	
			0.23-4.68				-0.90-8.36		1.00-1.05				0.99-1.09	
Coxarthrosis [arthrosis of hip]	M16	2,969												
… overweight/obesity mediated			2.25				4.08		1.02				1.04	
			0.19-3.85				-0.74-6,89		1.00-1.04				0.99-1.07	
Gonarthrosis [arthrosis of knee]	M17	3,762												
… overweight/obesity mediated			6.26				11.35		1.07				1.13	
			0.52-10.71			-2.06-19.16		1.01-1.12				0.98-1.24	
Alzheimer’s disease	G30	993												
… overweight/obesity mediated			0.75				1.36		1.01				1.01	
			0.04-1.94				-0.14-3.47		1.00-1.02				1.00-1.04	
… hypertension mediated			0.80		1.90				1.01		1.02			
			-0.06-3,07		0.34-4.88				1.00-1.03		1.00-1.05			
… diabetes mediated			0.05						1.00					
			0.01-0.12						1.00-1.00					
Sleep apnoea	G47.3	694												
… overweight/obesity mediated			7.62				13.80		1.08				1.16	
			0.63-13.03				-2.51-23.30		1.01-1.15				0.98-1.30	

**Fig 2:**
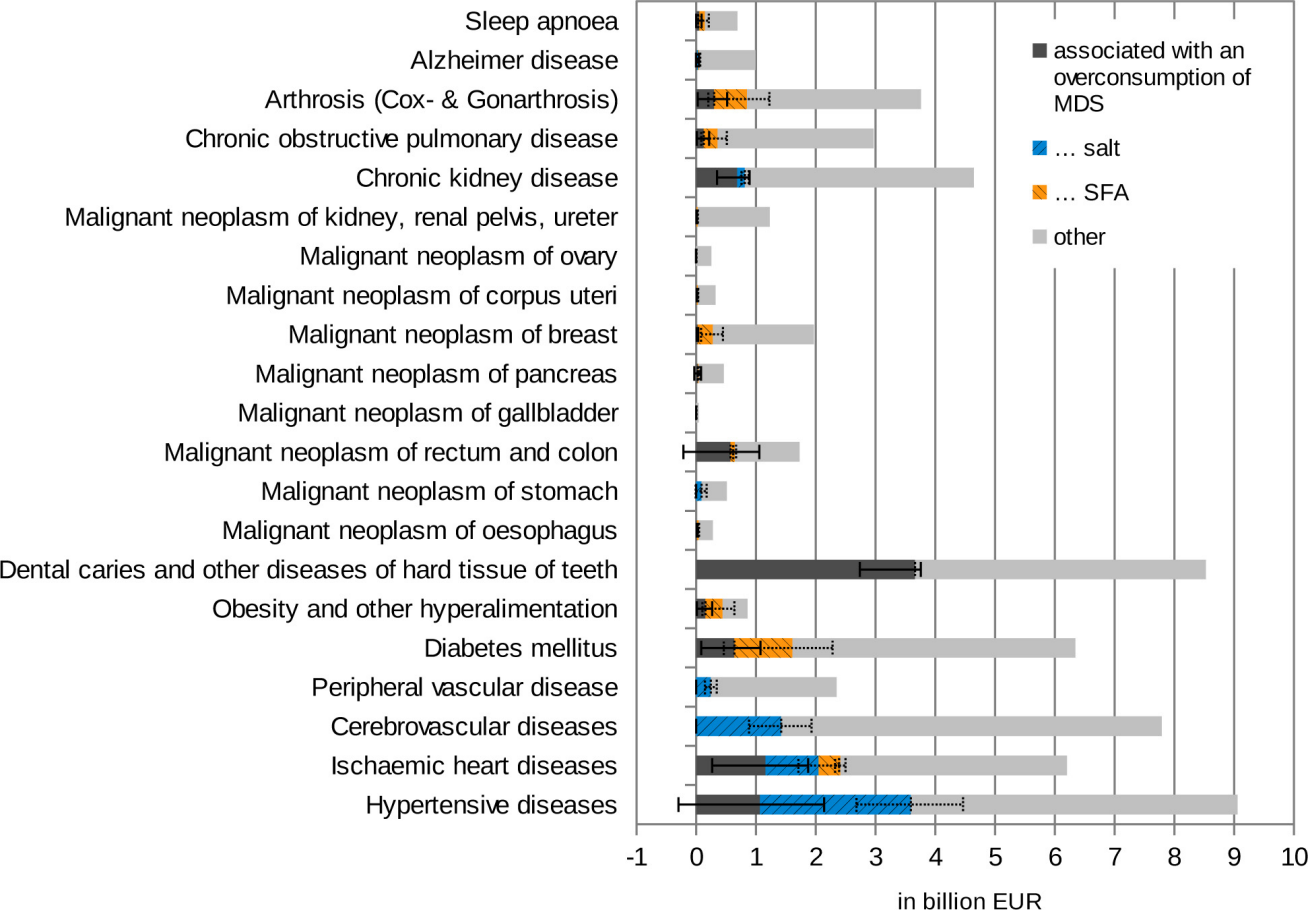
Healthcare costs associated with an overconsumption of MDS, salt and SFA according to diseases (incl. CI95%).

Fig 2 shows the specific attributable fraction of the excessive intake of MDS, salt and SFA for each of the diseases / disease groups considered. The highest shares of the total corresponding treatment costs were observed for (in descending order):

- obesity and other hyperalimentation (51%)
- dental caries and other diseases of the hard tissues of teeth (43%)
- hypertensive diseases (40%)
- ischemic heart diseases (39%)
- rectum and colon cancer (38%)
- diabetes mellitus (25%).

### Reduction scenarios and salt sensitivity analysis

**Fig 3:**
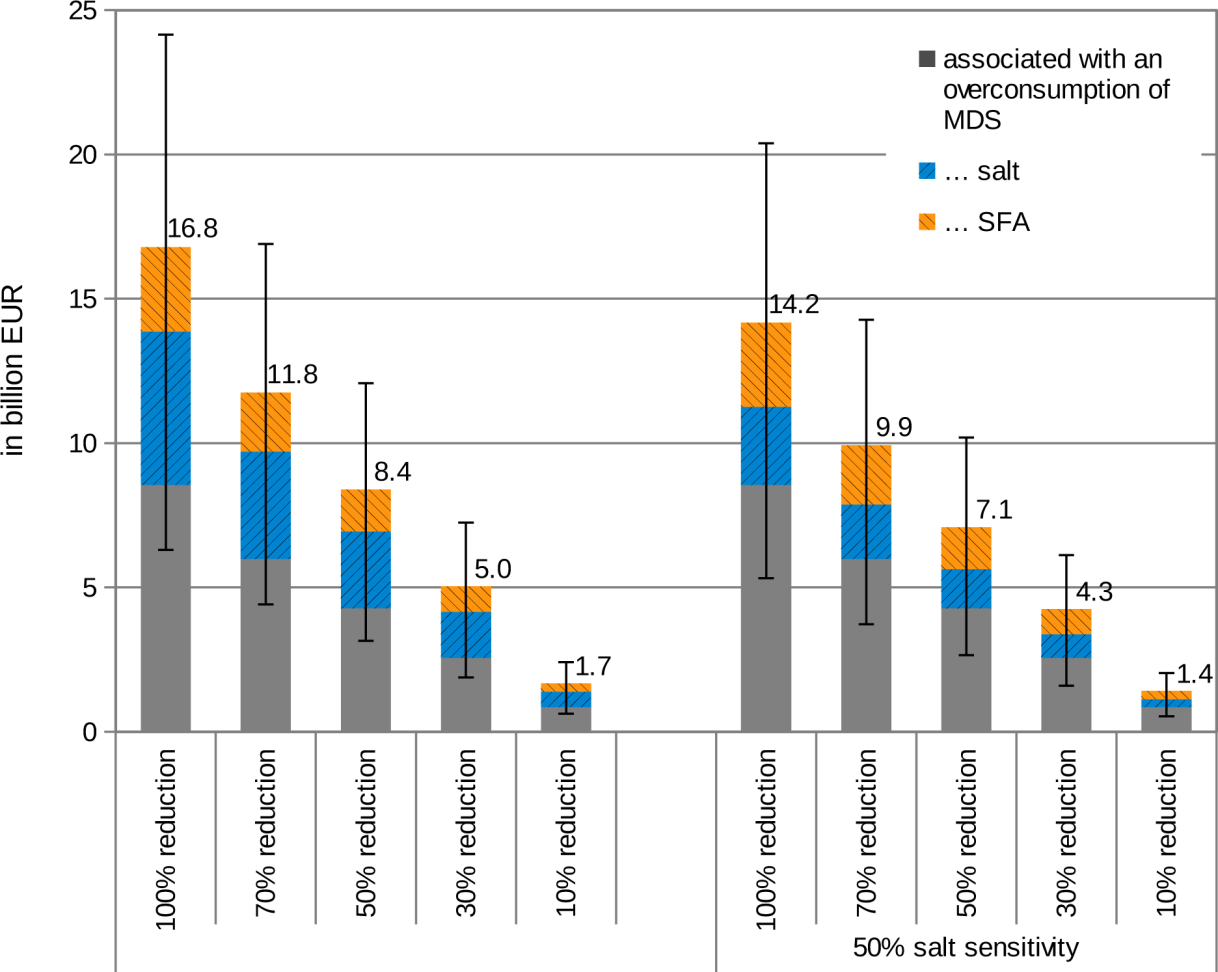
Predicted healthcare cost savings depending on different reduction levels of excessively consumed MDS, salt and SFA as well as the same scenario with a 50% salt sensitivity (incl. CI95%).

Fig 3 presents the results for different reduction scenarios. The first scenario, “100% reduction w/o 50% salt sensitivity”, builds upon the results presented in the previous chapter. In this scenario, a 100% reduction of the excessively consumed risk factors was assumed in the context of the official dietary recommendations. Given that a 100% reduction might be difficult to transfer at once into practice, different reduction scenarios were considered with a 10%, 30%, 50% and 70% reduction respectively. This reflects a gradual transfer more accurately. The expected healthcare cost savings vary between 1.7 billion EUR in the case of a 10% reduction (CI95%: 0.6–2.4) and 16.8 billion EUR in the case of a 100% reduction (CI95%: 6.3–24.1).

Further, Fig 3 shows the effect of 50% salt sensitivity concerning hypertension-related diseases in Germany according to [28]. With the exception of stomach cancer (ICD10-C16), 50% salt sensitivity was assumed for all remaining corresponding diseases (hypertensive diseases, ischemic heart diseases, cerebrovascular diseases, peripheral vascular diseases, chronic kidney disease and Alzheimer’s disease triggered by hypertension).

### Review and outlook of risk factor-related healthcare costs

**Fig 4:**
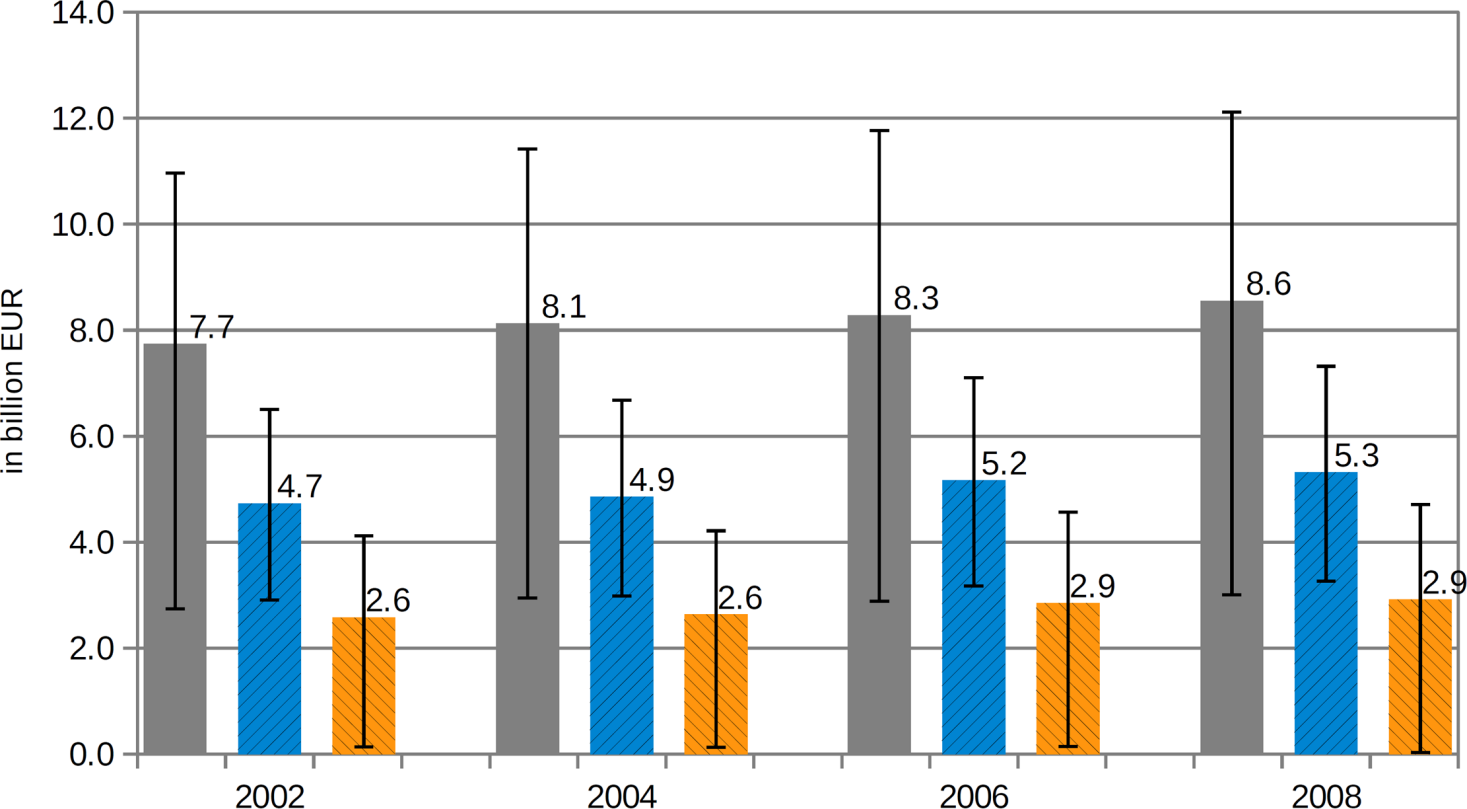
Attributable healthcare expenditure due to an excessive intake of MDS, salt and SFA from 2002 to 2008 (incl. 95%CI), single presentation.

Using the data concerning direct healthcare costs from the official Federal Health Monitoring for the years 2002, 2004 and 2006 [19] and applying the same methodological approach as described in the materials/methods section, the period from 2002 to 2008 was visualised (Fig 4). Comparing the years 2002 and 2008, the strongest increase—13.2%—was observed for diseases related to an excessive intake of SFA—mainly resulting from overweight/obesity-associated diabetes (+213 million EUR). Salt-related diseases showed an increase of 12.5% (mainly due to an increase of cerebrovascular and hypertensive diseases, totalling +295 million EUR and +276 million EUR respectively). The costs of MDS-associated diseases rose by just 10.5%, but by the greatest amount in absolute terms—814 million EUR. On average, all three of the risk factors considered led to an 11.6% rise in related healthcare costs. Nevertheless, this nominal increase has to be seen in the context of the corresponding inflation rate, which stood at 12.2% between 2002 and 2008 in Germany [57].

**Fig 5:**
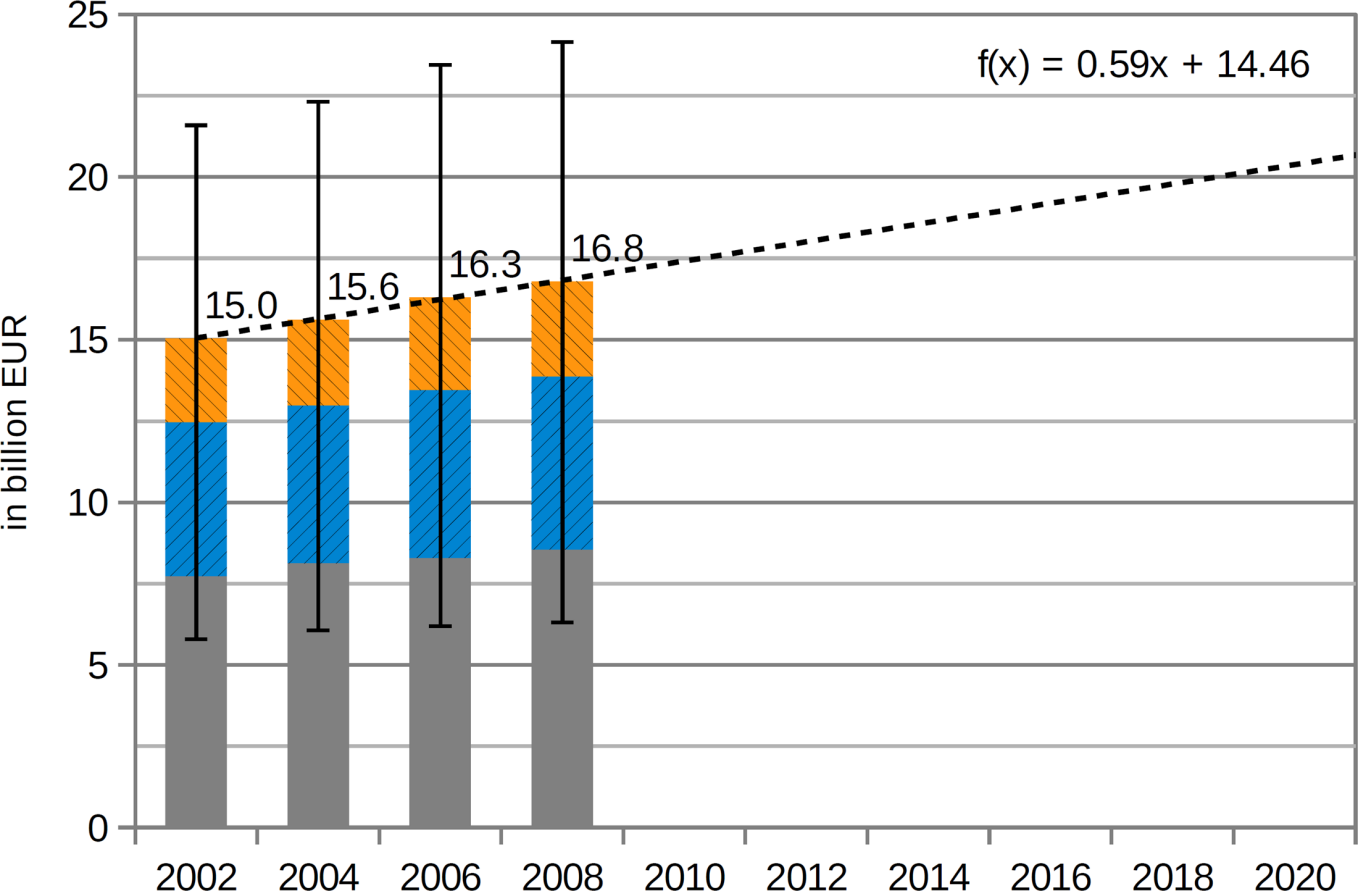
Attributable healthcare expenditure due to an excessive intake of MDS, salt and SFA from 2002 to 2008 (incl. 95%CI and regression towards 2020), aggregated presentation.

By using a linear regression function to extrapolate the trend observed from 2002 to 2008 until the year 2020, the economic burden of the excessive consumption of MDS, salt and SFA might exceed 20 billion EUR. Nevertheless, this trend would be mainly due to price inflation—see Fig 5.

## Discussion

As the literature search did not yield any studies with a similar goal and design, a direct comparison of the results generated is not possible. Nevertheless, comparative conclusions were drawn with respect to single diseases / disease groups considered in this and other studies. Compared to the study by Arab-Kohlmeier et al. (1993) [18], which put the contribution of unhealthy nutrition at a total of 33% of direct medical costs, this study identified a share of 7% (CI95% 2%-10%). The difference is mainly due to the fact that this study exclusively examined the specific risk factors MDS, salt and SFA, whereas the study by Arab-Kohlmeier et al. (1993) [18] was intended to consider a broad range of diet-related risk factors—considering also factors such as alcohol, cholesterol, different types of fatty acids, fibre, etc. For Switzerland, the direct medical costs of overweight/obesity and related comorbidities were estimated to be 2.3%-3.5% of total healthcare expenditure [15]. On a global level, in their meta-study Withrow and Alter (2010) calculated that overweight/obesity-related diseases were responsible for between 0.7% and 2.8% of corresponding healthcare expenditure [9]. In this study, a share of 1.4% (CI95%: -0.1%-1.8%) was identified, which can be explained by the fact that this study only considered MDS and SFA with respect to overweight/obesity. When considering these results, it must be remembered that SFA alone do not trigger overweight/obesity. SFA may only trigger overweight/obesity in the case of a positive energy balance, i.e. if more calories are ingested than metabolically burned. As shown in Table A in the S1 File (supporting information), for a physical activity level of 1.4 the average energy uptake of the German population (from 15 years onwards) was in line with the official recommendations. Moreover, it has to be stressed that, in addition to a positive energy balance and influencing lifestyle factors (like physical inactivity) a further factor can explain the prevailing overweight/obesity pandemic: antibiotics. Recent work by Ajslev et al. (2011) [58], Cho et al. (2012) [59] and Trasande et al. (2013) [60] has shown in animal and human trials that antibiotic treatments in early youth/childhood may stimulate overweight/obesity by way of an altered gut microbiome.

Further, as regards SFA there is increasing evidence that the impact of dietary saturated fat on cardiovascular diseases may be influenced by the food matrix through which the fatty acids are consumed. Cheese intake may not increase plasma cholesterol concentrations compared with butter of equal SFA content [61, 62]. This has been partly attributed to increased faecal fat excretion and the calcium content of these foods [63]. In the Multi-Ethnic Study of Atherosclerosis [64], a higher consumption of SFA from dairy was associated with a lower CVD risk, higher SFA from meat was associated with a higher risk of CVD, while SFA from butter, plant, or mixed sources showed no relationship. In this context, when designing new fat substitutes it appears most important that the substitutes meet the haptic and technological requirements of meat products. These food-specific aspects may explain the results of Chowdhury et al. (2014) [65], who argue in favour of a revision of the current dietary recommendations for saturated fat (see also [66] and [67]).

It should also be kept in mind that the kind of substitute has an effect on etiological processes. Mensink et al. (2003) have shown that substituting saturated fatty acids (SFA) with poly-unsaturated fatty acids (PUFAs) is most beneficial with regard to ischemic heart diseases, followed by mono-unsaturated fatty acids (MUFAs), where the effect is slightly smaller. Substituting SFA with carbohydrates, on the other hand, has shown no significant effect [37]. In order to derive specific effect sizes as regards the current total and HDL cholesterol levels in Germany (as an assumed risk factor for developing ischemic heart diseases), this study used data from the last DEGS1 study (2008–2011) [68].

As regards salt consumption and the prevalence of hypertension, Palar and Sturm (2009) [69] calculated $18 billion of healthcare expenditure in the US that could be saved with a reduced daily average intake of 2300 mg sodium per person (based on the National Health and Nutrition Examination Survey 1999–2004). With a share of 1.5% of the total national health-care costs, this result is comparable to the 2.0% identified in our study.

Whenever possible, this study used current population- and outcome-specific etiological effect sizes per unit of exposure based on high-quality epidemiological or econometrical studies (e.g. IHME 2014 [32], Mensink et al. 2013 [70]), or it adapted these to the corresponding consumption level in Germany (e.g. Arnold et al. 2014 [38], Basu et al. 2013 [46], Mensink et al. 2003 [37], Moynihan & Kelly 2013 [47]). If such effect sizes were not available, less current and less population- and context-specific effect sizes had to be used in order to perform the computational modelling. However, as mentioned in the materials/methods section, sensitivity and uncertainty analyses were conducted in order to visualise related impacts on the study results. With regard to MDS and salt, the results obtained can be considered to be robust, as the lower and upper bounds of the 95% confidence intervals indicate clear positive results. In the case of SFA, the reliability is limited since the lower bound of the CI95% is close to zero (with a possible cost saving of just 32 million EUR per year, which equals to 0.0001% of medical costs in Germany).

## Conclusions

As investigated in this study, an association of dietary factors (in particular an excessive intake of MDS, salt and SFA) and of clinical endpoints with related treatment costs exists for a broad set of diseases, with direct healthcare costs totalling 16.8 billion EUR in Germany (CI95%: 6.3–24.1 billion EUR). In other words: These costs could be saved if people’s intake of MDS, salt and SFA were adequate. However, since this study only considered direct medical treatment costs related to an adequate intake, the actual potential societal and economic gains—resulting both from direct and indirect cost savings—may be higher than 16.8 billion EUR. Moreover, against the backdrop of a steadily ageing society and the foreseeable increases in disease burdens over the coming decades, this figure may well rise (not only nominally, but also when adjusted for inflation).

It follows that measures aimed at optimizing diets and recipes could be used as effective leverage in order to relieve pressure on healthcare, health insurance and national tax levy systems. Health insurance companies, governmental institutions, but also commercial enterprises, which should have an intrinsic interest in the health of their employees and customers, will therefore be required to develop proper incentive mechanisms that facilitate healthier nutrition. In particular, companies in the food, beverage and catering industries should be supported in their efforts to optimize existing recipes, and develop new ones (possibly by introducing new components) with enhanced nutritive performance, allowing all customers easier and healthier food choices.

With regard to a possible fat substitution, it has to be kept in mind that the food matrix may have an influence on the nutritive impact of the specific component. With this in mind, when designing new fat substitutes it seems most important that substitutes meet the haptic and technological requirements of meat products (see discussion). Furthermore, when compared to SFAs, poly-unsaturated fatty acids (PUFAs) showed the strongest risk-decreasing effect for developing ischemic heart diseases, followed by mono-unsaturated fatty acids (MUFAs) and carbohydrates.

As far as a possible salt substitution is concerned, trade-offs must be considered with respect to an adequate iodine supply. Presently, more than 50% of the average iodine supply in Germany occurs via the intake of common table salt [20]. So if salt substitutes with a better nutritive profile are to be used in food products, it should be possible to enrich them with iodine.

The findings of this study confirm the imperative of the UN High-Level Review on Non-Communicable Diseases, in which governments agreed to intensify and accelerate efforts towards creating a world free of the avoidable burden of NCDs [71]. The High-Level Review is based on the NCD Global Monitoring Framework (WHO, 2013) [72], which identified a set of nine core targets to minimize the impacts of NCDs by 2025. Four out of these nine core targets can be addressed by a balanced consumption of salt (targets 1, 4, 6 and 8), whereas target 7 (“Halt the rise in diabetes & obesity”) as well as target 1 (“A 25% relative reduction in the overall mortality from NCDs”) can be achieved by a balanced intake of MDS and SFA.

## Supporting Information

S1 File Table A: Intake and D-A-CH reference values of the considered risk factors according to population groups. Table B: Results of the literature search. Table C: List of excluded studies. (DOC)
