# Supplementary material for: Healthcare Costs Associated with an Adequate Intake of Sugars, Salt and Saturated Fat in Germany: A Health Econometrical Analysis
Source: PLoS One. 2015 Sep 9;10(9):e0135990. doi: 10.1371/journal.pone.0135990 (PMC4566993; doi:10.1371/journal.pone.0135990)
Supplement: S1 File — (DOC) [file pone.0135990.s001.doc]

**Supporting information**

Healthcare costs associated with an adequate intake of sugars, salt and saturated fat in Germany: a health econometrical analysis

Toni Meier^1^*, Karolin Senftleben^1^, Peter Deumelandt^1^, Olaf Christen^1^, Katja Riedel^2^, Martin Langer^2^

^1^ Institute of Agricultural and Nutritional Sciences, Martin Luther University Halle-Wittenberg, Halle (Saale), Germany

^2^ BRAIN Biotechnology Research And Information Network AG, Zwingenberg, Germany

* Corresponding author

Email: [toni.meier@landw.uni-halle.de](mailto:toni.meier@landw.uni-halle.de) (TM)

Table A Intake and D-A-CH reference values of the considered risk factors due to population groups

NNSII … National Nutrition Survey II

PAL … Physical Activity Level

Table B Results of the literature search (November 2014)

Table B Results of the literature search (November 2014) (continuation)

Table B Results of the literature search (November 2014) (continuation)

Table C List of excluded studies (after in-depth examination)

**Table C List of excluded studies (after in-depth examination)(continuation)**

**Table C List of excluded studies (after in-depth examination) (continuation)**
